# Supplementary material for: Ecological dynamics of three persistent opportunistic pathogens in hospital sinks and their potential antagonistic bacteria
Source: mSystems. 2026 Feb 4;11(3):e01546-25. doi: 10.1128/msystems.01546-25 (PMC13011461; doi:10.1128/msystems.01546-25)
Supplement: Supplemental figures — Figures S1 to S4. [file msystems.01546-25-s0001.docx]

**Supplemental figures - Bourdin et al., 2025 (Ecological dynamics of three persistent opportunistic pathogens in hospital sinks and their potential antagonistic bacteria)**


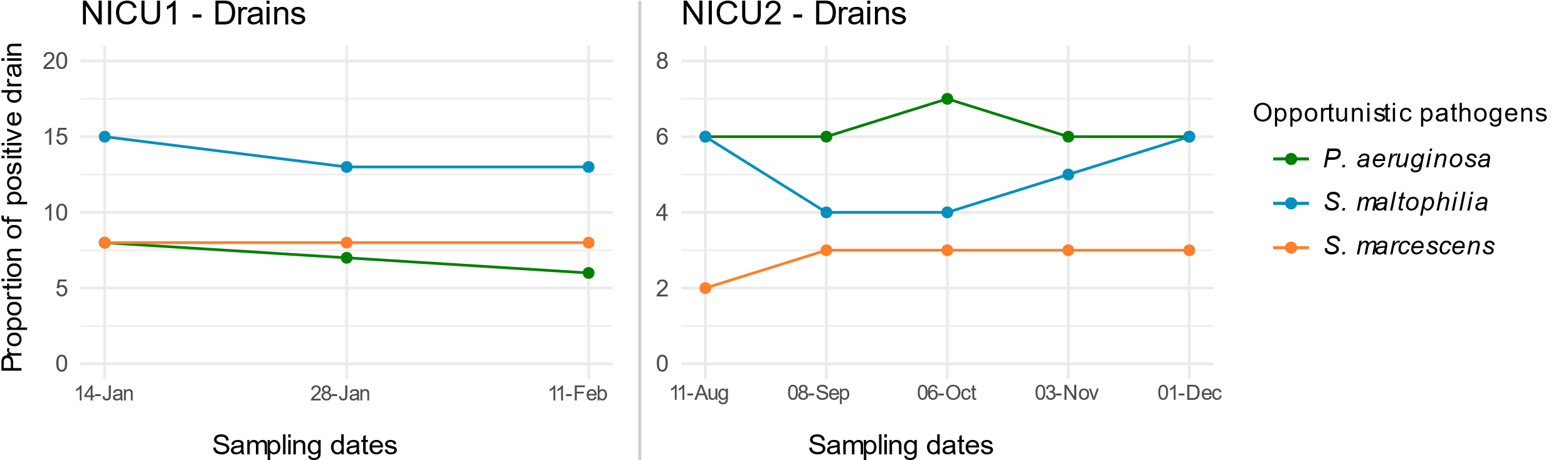


**Figure S1. Number of faucets and drains positive for *P. aeruginosa*, *S. marcescens*, and *S. maltophilia*, depending on sampling dates in 2020.** A total of 20 sinks were sampled in NICU#1 (charts on the left), and 8 sinks were sampled in NICU#2 (charts on the right).


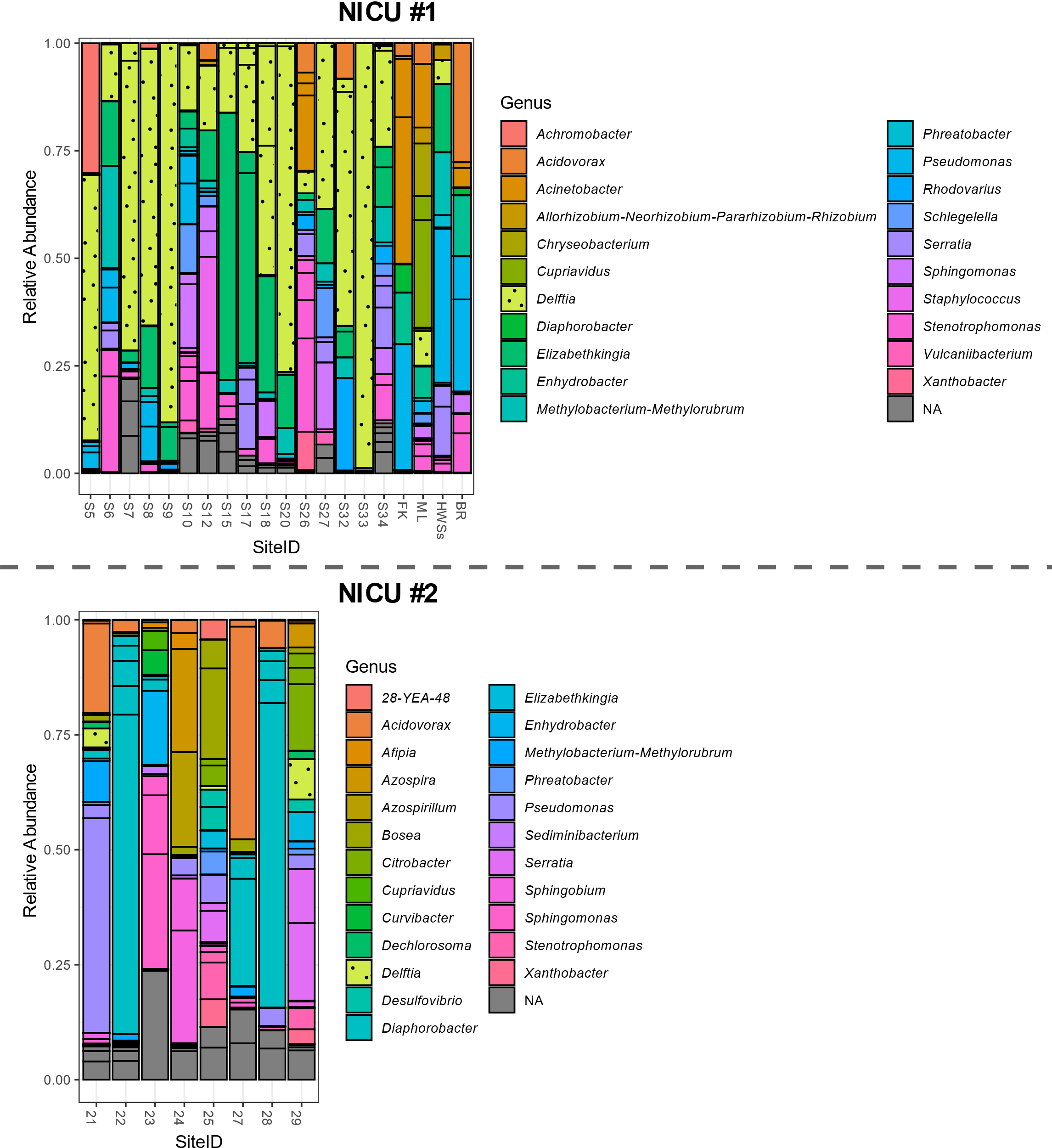


**Figure S2. Average relative abundance of bacterial genera found in sink drains in both NICUs.** Each relative abundance results from the average ASV abundance over several sampling dates.


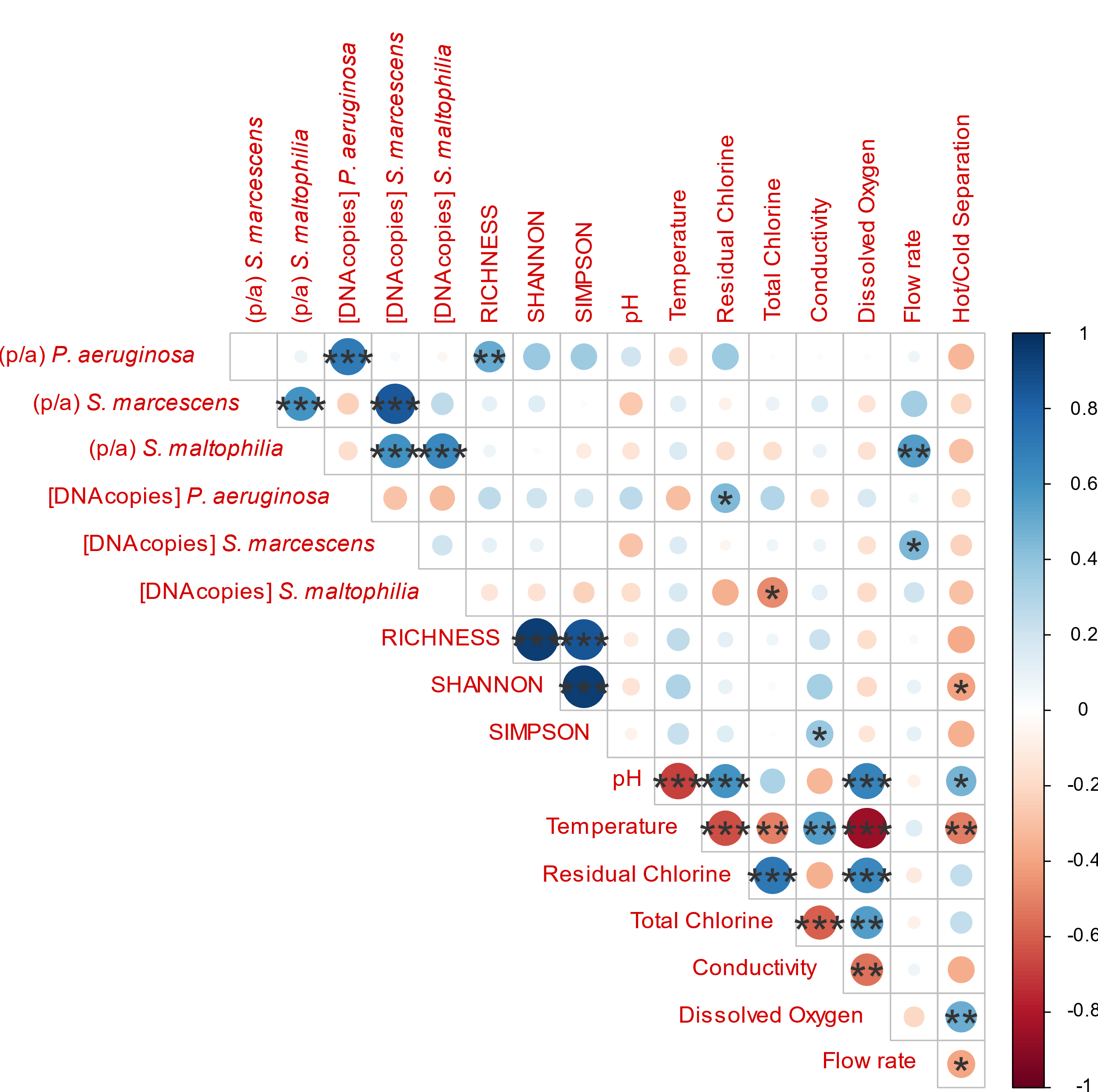


**Figure S3. Spearman correlations illustrate the associations between opportunistic pathogens and both biotic and abiotic parameters in sinks, from both NICU.** Pathogen “presence/absence” (p/a) and concentration (DNA copies per milliliter) are paired with alpha diversity indices (Richness, Shannon Index, and Simpson index), along with physico-chemical parameters of tap water measured at sink faucets. Circles are color-coded to signify the direction of correlation: blue for a positive correlation, red for a negative one. Stars denote *p* values for Spearman correlations: (*) for *p* < 0.05, (**) for *p* < 0.01, and (***) for *p* < 0.001.

**
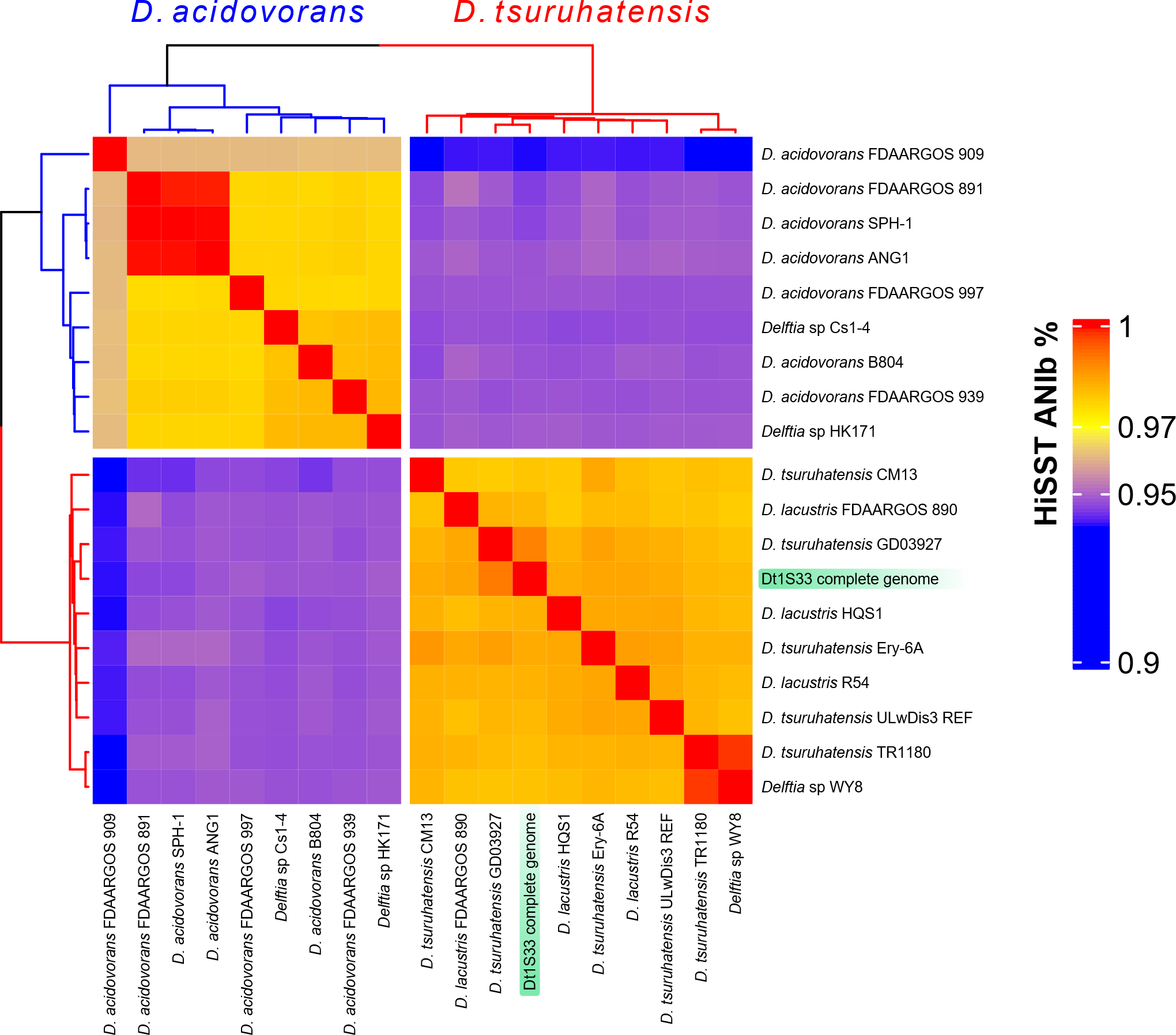
**

**Figure S4. Taxonomic analysis of *Delftia* sp. strain discrimination through whole genome sequence comparison.** The heatmap illustrates the Average Nucleotide Identity based on BLAST (ANIb) scores. The data encompasses whole genomes obtained from NCBI's GenBank database (<https://www.ncbi.nlm.nih.gov/genbank/>) and includes the Dt1S33 strain isolated in this study.
