# Supplementary material for: Ecological dynamics of three persistent opportunistic pathogens in hospital sinks and their potential antagonistic bacteria
Source: mSystems. 2026 Feb 4;11(3):e01546-25. doi: 10.1128/msystems.01546-25 (PMC13011461; doi:10.1128/msystems.01546-25)
Supplement: Supplemental Methods — Details of NICU environment and sink sampling, microbial detection and genotyping, sequencing approaches, bacterial transformation and antagonism assays, data accession, and study limitations. [file msystems.01546-25-s0002.docx]

Supplementary methods, Bourdin et al., “Ecological dynamics of three persistent opportunistic pathogens in hospital sinks and their potential antagonistic bacteria”

# NICU description

NICUs 1 and 2 are two structurally and operationally distinct units situated in different hospitals in Montreal.

NICU#1 was built in 2015 and has a capacity of 35 beds with mainly single rooms and 5 double rooms. Double rooms had the same configuration as single rooms, with two sinks but without a separation between beds. Sink design was uniform. Sampled sinks were mostly located at patient room entrance (n = 16), separated from the corridor by a sliding door. A self-disinfection drain was installed before the study on a sink (1-S10). Four other sinks included in this study were localized outside of the patient rooms: at the entrance of the NICU (handwashing-station 1-HWSs), in the family kitchen (1-FK), in the breastfeeding room (1-BR), and in the milk laboratory (1-ML). Sink configuration in NICU#1 consisted of hot and cold water activated through two distinct pedals, copper and stainless-steel copper plumbing, with the faucet not aligned with the sink drain inlet, and P-Traps in chrome-plated brass. The water drainage system of the NICU floor was mainly vertical, connected directly to the main sewage collectors on the lower floors. Overall, 13 out of 20 rooms were connected to one or two other sinks from adjacent rooms before being connected to the main vertical drain.

NICU #2, built before 2000, has a different layout with all patients in one large room and a capacity of 16 beds. A total of eight sinks were sampled, including sinks 2-S22, -S24, -S25, -S28 that are scattered around the same large room where patients are located. The sink 2-S21 was in a separate bathroom, 2-S23 in a lactation room, 2-S29 at the NICU entrance and, just behind, 2-S27 in a rest room for healthcare staff. Sinks in NICU#2 were composed of different designs, varying in terms of the presence of aerators (which affect the tap water flow rate), the presence of mixing valves or separate hot/cold water inlets. Furthermore, the depth of drains differed between sinks in NICU#2. Sink drain materials also varied, including stainless steel, copper, and PVC.

# Sink drain sampling and processing

The comprehensive sampling procedures are detailed in our previous study (1). Briefly, 100 mL of drain water was collected using a sterilized plastic tube attached to a syringe, while sink biofilms were sampled using a nylon-flocked swab (Puritan Medical Products, Guilford, ME, USA) extended with a rod. Blank samples (2 mL PBS in 15 mL tubes) were prepared before each sampling routine to verify sterile conditions. A defined volume of tap water (1L, hot and cold water, 1:1) was also collected. No growth was observed for quality control samples after 48 h of incubation at 30°C or 42°C, depending on the selective culture medium. Samples were processed within six hours. In NICU#2, drain biofilm samples were combined with drain water samples due to similarities found in pathogen prevalence compared to NICU#1, and to simplify the sample processing.

Sinks were tested for the presence of three opportunistic pathogens (OPs): *P. aeruginosa*, *S. marcescens* and *S. maltophilia*. Direct analysis of environmental genomic DNA (eDNA) was performed for drain water (WD), drain biofilm (BD), and faucet water (WF) samples. An initial cultivation step on selective media was used for faucet aerator biofilm (BF) and WF samples due to the lower level of contamination.

Samples were processed for downstream isolation efforts, PCR analyses or archive storage. Briefly, tap and drain water samples (500 mL and 25-50 mL, respectively) were filtered on 0.45 µm pore size mixed cellulose ester (MCE) sterile membranes (Millipore Sigma-Aldrich, Oakville, ON, Canada). Membranes were put on selective media, supplemented with antibiotics, depending on the species targeted: Sm-MacConkey medium (incubated during 48 h at 30°C) for *S. marcescens* (1) ; nalidixic acid cetrimide medium (48h at 42°C) for *P. aeruginosa* (2) ; and mSM2I medium (48h at 30°C) for *S. maltophilia*. The mSM2I medium, a modified SM2I medium (3), has been adapted to our needs as detailed in our forthcoming publication (4). A detailed description of subsequent sample processing and DNA extraction procedures is provided in our previous study (1).

Tap water physicochemical parameters were systematically monitored during each sampling, encompassing measurements of flow rate, temperature (Omega Engineering, Norwalk, CT, USA), pH, conductivity, dissolved oxygen (HQ4300 Portable Multi-Meter, HACH, London, ON, Canada), as well as total and residual chlorine (pocket colorimeter from HACH, London, ON, Canada). Drain water turbidity (HACH, London, ON, Canada) was also measured.

# Sink usage frequency estimation

Sink usage frequencies were estimated by measuring temperature fluctuations every 30 seconds at the hot and cold tap water inlet, using temperature surface sensors (Surface Thermocouple with Self-Adhesive Backing, Omega, St-Eustache, QC, Canada) connected to data loggers (Portable Thermometer Thermocouple Data Loggers with SD Card, Omega, St-Eustache, QC, Canada). Only a subset of sinks from NICU#1 was examined, encompassing 3 sinks situated in common areas (1-ML, -BR, -HWSs) and an additional seven sinks chosen at random from patient rooms in NICU#1 (sinks 1-S5, -S6, -S18, -S20, -S27, -S32, -S34). Analysis was done on RStudio environment by measuring the average of peak/valley of temperature series (minimum peak/valley threshold at 1°C), using “findPeaks” function for hot water inlet series, or “findValleys” function for cold water inlet series, available in the package *quantmod* (5).

# Detection and genotyping of the OPs

Presumptive identification of isolate species was performed by PCR targeting the three or four loci of corresponding HiSST schemes (4, 6). Occurrence of each OP in eDNA samples was primary screened by PCR targeting only one locus of the corresponding HiSST schemes to reduce manipulation effort, including *bssA* for *S. marcescens* detection, *pheT* for *P. aeruginosa* and *glnG* for *S. matophilia* as described previously (1, 4). Depending on the sample type (i.e., isolates or samples from faucets or drains), between 50 and 70 libraries were combined at equimolar proportion. Then, library pools were sequenced at the Centre d'expertise et de services Génome Québec (Montréal, Canada), using Illumina MiSeq PE-250 platform (100,000 sequences were allocated per libraries pool). The entire raw sequencing reads processing was performed using the DADA2 pipeline (7) adapted for HiSST schemes (4), using the R script "Script_RUN_FunHiSSTDada2.R" and "FunHiSSTDada2.R" function available on the dedicated GitHub repository (<https://github.com/LaboPC/HiSST-schemes_TB>). The pipeline encompassed primer sequences removal with the software Cutadapt v. 2.10 (8), default parameters specified in the package dada2 v1.8.0 (7) including error correction, denoising, and paired ends merging, and additional steps of taxonomic assignment and for HiSST analysis. Only non-chimeric, and highly specific amplicon sequence variants (ASV, filtered sequences displaying 100% identity) for targeted species, were retained for subsequent analysis, based on the amplicon length and BLAST identity percentage against HiSST databases (4). Barcoded primers used for libraries preparation, and the proportion of reads remaining after each step of the DADA2 pipeline are provided in Tables S1 to S4.

# Quantification of OPs

Three distinct sampling dates were screened within both NICUs. This encompassed all the sampling dates for the NICU#1 campaign, as well as a subset of three out of five sampling dates within NICU#2. This subset included the initial, intermediate, and final dates (samples labeled as “0224”, “0280” and “0336” within NICU#2 samples). PCR conditions were optimized for each HiSST locus used (i.e., temperature gradient for primer hybridization, DNA dilution), including primer pairs targeting the *bssA* locus for *S. marcescens* quantification, the *glnG* locus for *S. maltophilia*, and the *pheT* locus for *P. aeruginosa*. The dilution factor for DNA extracts was preliminary determined by estimating the intensity of PCR amplification. This assessment was carried out under UV light on a 1.5% agarose gel after the primary OPs screening. Dilution factors were categorized into three intensity levels: 1/1000, 1/100 or 1/10 dilutions for strong, moderate or weak amplification, respectively. Individual adjustment of the DNA dilution was carried out for samples that did not pass the quality controls of ddPCR.

Simplex PCR was conducted for *P. aeruginosa* quantification using primers designed to target the *pheT* locus, each at a final concentration of 100 nM. Duplex PCR was optimized to achieve simultaneous quantification of both *S. marcescens* and *S. maltophilia*, targeting the *bssA* locus (primers at 100 nM each) and the *glnG* locus (primers at 250 nM each), respectively. The 21 µL reaction mixes contained 10.5 μL of EvaGreen commercial mix (2x QX200™ ddPCR™ EvaGreen® Supermix, Bio-Rad Laboratories, CA), 1.05 μL of each forward and reverse primers, 5 μL of diluted DNA (as described in the previous paragraph), supplemented with Milli-Q® ultrapure water. Microdroplets were generated and emulsified samples were transferred to 96-well PCR plates following the guidelines provided in the manufacturer's protocol (Instruction Manual, QX200™ Droplet Generator, Bio-Rad). Optimum conditions for PCR cycles included enzymatic activation at 95°C for 5 min, followed by 50 cycles of 30 s amplification at 95°C, 1 min hybridization at 62°C, and 30 s elongation at 72°C. Finally, enzymatic inactivation included 5 min at 4°C followed by 5 min at 90°C. Quality control was applied to each sample, ensuring that more than 10,000 microdroplets were obtained per reaction, a variation in minimum and maximum threshold of less than 10% between negative and positive microdroplets, and DNA copy concentration greater than 2 copies / μL. Samples with DNA concentrations between 1 and 2 copies / μL were quantified by ddPCR in duplicates. The raw DNA copy concentration per microliter of each sample was calculated taking into account the dilution factors applied during ddPCR steps, dilution of the samples to 25 ng/µL after elution of the extracted DNA, re-suspension of the DNA after extraction in 50 µL of water, and the volume of drain water actually filtered (the volume varied from 25 to 50 mL depending on the sample and on the clogging of the MCE membranes during filtration) (Table S5).

# Bacterial transformation

*P. aeruginosa* strain UCBPP-PA14 was tagged using two plasmids transformed in the cells by electroporation as previously described (12). The pUC18-mini-Tn7T-*lux* plasmid carrying the *luxCDABE* operon was co-transformed with the pTNS3 helper plasmid encoding the Tn7 site-specific transposition pathway (Table 1). Transformed cells were subsequently selected on LB agar plates supplemented with 20 µg/mL gentamicin. The resulting PA14 strain is referred to as PA14-*lux.*

A similar approach was applied to *S. maltophilia* strain 810-2 (=ATCC 13637, =LMG 958, =NCTC 10257; CP008838). In this case, the pUC18-mini-Tn7-GFPmut3 plasmid containing a GFP reporter and the pTNS3 plasmid were transformed into the cells as before with some modifications (Table1). Cells were washed with 10% glycerol and transformants were selected on 100 µg/mL gentamicin (13). The tagged strain is referred as SM810-GFP.

Table 1: Plasmids used in this study

| Plasmid | Description | Source |
| --- | --- | --- |
| pTNS3 | Plasmid expressing a Tn7 transposase (*tnsABCD*) from P1 and P*_lac_* , ampicillin resistant | (14) |
| pUC18-mini-Tn7T-*lux* | Chromosomic integration vector pUC18-mini-Tn7T with *luxCDABE* operon, gentamicin resistant | (15) |
| pUC18-mini-Tn7-GFPmut3 | Chromosomic integration vector pUCP18-mini-Tn7 with a GFP transcriptional fusion, gentamicin resistant | (16) |

# In-vitro tests of a potential bacterial antagonist against OPs

The antagonistic potential of *D. tsuruhatensis* Dt1S33 towards both *P. aeruginosa* (PA14-*lux*) and *S. maltophilia* (SM810-GFP) was investigated in the context of biofilm formation. In the case of *P. aeruginosa*, bacterial suspensions were prepared with the following ratios of Dt1S33 to PA14-*lux*: 1:1, 2:1, 4:1. For this experiment, 10% TSB medium containing 0.5% casamino acids (CAA) was used. A control with only PA14-*lux* or SM810-GFP was also prepared. Subsequently, 200 μL of each suspension was distributed in the wells of a white 96-well plate. Each suspension was added in six separate wells. The plate was then incubated without agitation for 24h at room temperature (22°C). The following day, planktonic bacteria were removed, and fresh medium was added to the wells. Luminescence, indicative of the overall presence of PA14-*lux* cells within the biofilm, was quantified using a Cytation3 microplate reader (BioTek, Winooski, VT, USA). The same method was applied for *S. maltophilia* with small modifications. LB medium was used, and suspensions were deposited in the wells of a black 96-well plate. Following a 24 h incubation at 22°C, the plate was washed to remove planktonic bacteria and fresh medium was added to each well. Fluorescence corresponding to the growth of SM810-GFP in biofilms was measured using the Cytation3 (489 nm/520 nm). To validate specificity against OP strains, we replicated the procedure, substituting *D. tsuruhatensis* Dt1S33 with *Burkholderia cenocepacia* K56-2 ([SAMN14693155](https://www.ncbi.nlm.nih.gov/biosample/SAMN14693155)).

# Whole genome sequencing

The whole genomes of three isolates were sequenced: *D. tsuruhatensis* Dt1S33 (lab collection ED4661), *S. marcescens* ED4677 (= sample BWD-29-0280-Sm1, *S. marcescens* strain corresponding to the dominant NICU#2 genotype), *S. maltophilia* ED4675 (= sample BF-24-0280-St2, *S. maltophilia* strain isolated in sink drain). Short-read WGS were performed with the Illumina NextSeq 550 platform at the Microbial Genome Sequencing Center (Pittsburgh, PA, USA). For *S. marcescens* ED4677 and *S. maltophilia* ED4675 isolates, the complete short-read assembly procedure is described (4).The *D. tsuruhatensis* Dt1S33 genome was assembled using Oxford Nanopore and Illumina to obtain a high-quality assembly genome (17), with the same isolate DNA extract used for Illumina WGS. ONT library was prepared following the protocol for the SQK-NBD114 Native Barcoding Kit 24 V14 (Oxford Nanopore Technologies), and sequenced on MinION R10.4.1 flow cells (FLO-MIN114). ONT read sets were basecalled and demultiplexed using Guppy software v6.4.6 with high accuracy model. Quality control filtering was performed using Filtlong (18) to remove the worst 10% of reads and short reads (< 5kb). Hybrid assembly of the genome was performed using Trycycler v0.5.4 (19) with 12 read sets and the assemblers Flye (20), Miniasm+Minipolish (21) and Raven (22). Trycycler's consensus sequence was polished using Medaka (23). Finally, after a quality control on Illumina reads using fastp (24), genome assembly was further improved using the short-read polishers Polypolish (25) and POLCA (26). The assembled genome comprises 6,542,007 base pairs, with a genome coverage of 300x. *S. marcescens* ED4677 (i.e., BWD-29-0280-Sm1) contigs and *D. tsuruhatensis* Dt1S33 complete genome were annotated using the RAST server (27) on the SEED environment (28) (Table S7).

# Accession numbers.

Raw sequencing reads have been deposited in the Sequence Read Archive of the NCBI in the BioProject [PRJNA1042964](https://www.ncbi.nlm.nih.gov/bioproject/PRJNA1042964). Assembled genomes of isolates *D. tsuruhatensis* Dt1S33 ([SAMN46474190](https://www.ncbi.nlm.nih.gov/biosample/SAMN46474190/)), *S. marcescens* ED4677 ([SAMN46474191](https://www.ncbi.nlm.nih.gov/biosample/?term=SAMN46474191)) and *S. maltophilia* ED4675 ([SAMN46474192](https://www.ncbi.nlm.nih.gov/biosample/?term=SAMN46474192)) have been deposited in the same BioProject. Other genomes used in this study were previously published (4), including *S. maltophilia* isolates (WF-25-0224-St, BF-25-0308-St) and *P. aeruginosa* isolates (BWD-25-0280-Pa, BF-HWSs-0021-Pa, WF-HWSs-0049-Pa).

# Limitations

This study relies on samples collected from specific sinks within two NICUs from the same city, albeit in different hospitals, potentially sharing similar hospital practices and a common source of potable water. Consequently, the extrapolation of our findings to other systems is challenging. Given the markedly distinct designs of the two NICUs – one characterized by modern units with separate patient rooms and the other by an older unit where all newborns are accommodated in a shared space – a broad spectrum of sink designs could be tested. Despite extensive sampling efforts, the limited number of sinks available constrained the ability to determine the primary factors influencing pathogen presence in drains. Moreover, the relatively small dataset, focused on only three species, limits the power to identify key drivers of variation across environmental and microbial factors. Other potential factors, such as the hygiene practices of healthcare personnel and other handling and maintenance measures that might influence the bacterial composition of sink drains, are not considered. These factors could potentially impact the presence of OPs. Handwashing was strictly enforced on both units, due to the epidemic situation and the shared patient room. Future studies could broaden their sampling scopes to include other healthcare units and further explore interactions between bacterial communities, OPs, and environmental factors.

Another limitation inherent to our methodology concerns the targeting of environmental DNA sequences within drain water and biofilms. These sequences can persist for several days, potentially leading to false-positive results in scenarios where no viable bacteria remain (29–31). However, with one exception, sinks were extensively used by healthcare workers, reducing relic DNA through regular P-trap flushing. In addition, samples were collected bi-weekly in NICU#1 and monthly in NICU#2 for HiSST analysis, minimizing the chance of relic DNA detection. Furthermore, HiSST analyses involves targeting multiple loci and a dedicated pipeline to remove non-specific sequences.

References:

1. Bourdin T, Benoit M-È, Monnier A, Bédard E, Prévost M, Charron D, Audy N, Gravel S, Sicard M, Quach C, Déziel E, Constant P. 2023. *Serratia marcescens* colonization in a neonatal intensive care unit has multiple sources, with sink drains as a major reservoir. Appl Environ Microbiol 89:e00105-23.

2. Goto S, Enomoto S. 1970. Nalidixic Acid Cetrimide Agar: A New Selective Plating Medium for the Selective Isolation of *Pseudomonas aeruginosa*. Jpn J Microbiol 14:65–72.

3. Adjidé CC, De Meyer A, Weyer M, Obin O, Lamory F, Lesueur C, Trouillet L, Biendo M, Ganry O, Eb F. 2010. La mise au point d’un milieu sensible, spécifique et prédictif de recherche de *Stenotrophomonas maltophilia* dans l’environnement des soins. Pathol Biol 58:11–17.

4. Bourdin T, Benoit M-È, Bédard E, Prévost M, Quach C, Déziel E, Constant P. 2024. High-throughput short sequence typing schemes for *Pseudomonas aeruginosa* and *Stenotrophomonas maltophilia* pure culture and environmental DNA. 1. Microorganisms 12:48.

5. Ryan JA, Ulrich JM, Smith EB, Thielen W, Teetor P, Bronder S. 2023. quantmod: quantitative financial modelling framework (0.4.24).

6. Bourdin T, Monnier A, Benoit M-È, Bédard E, Prévost M, Quach C, Déziel E, Constant P. 2021. A high-throughput short sequence typing scheme for *Serratia marcescens* pure culture and environmental DNA. Appl Environ Microbiol 87:e01399-21.

7. Callahan BJ, McMurdie PJ, Rosen MJ, Han AW, Johnson AJA, Holmes SP. 2016. DADA2: High-resolution sample inference from Illumina amplicon data. 7. Nat Methods 13:581–583.

8. Martin M. 2011. Cutadapt removes adapter sequences from high-throughput sequencing reads. 1. EMBnet.journal 17:10–12.

9. Herlemann DP, Labrenz M, Jürgens K, Bertilsson S, Waniek JJ, Andersson AF. 2011. Transitions in bacterial communities along the 2000 km salinity gradient of the Baltic Sea. 10. ISME J 5:1571–1579.

10. Klindworth A, Pruesse E, Schweer T, Peplies J, Quast C, Horn M, Glöckner FO. 2013. Evaluation of general 16S ribosomal RNA gene PCR primers for classical and next-generation sequencing-based diversity studies. Nucleic Acids Res 41:e1.

11. Quast C, Pruesse E, Yilmaz P, Gerken J, Schweer T, Yarza P, Peplies J, Glöckner FO. 2013. The SILVA ribosomal RNA gene database project: improved data processing and web-based tools. Nucleic Acids Res 41:D590–D596.

12. Choi K-H, Kumar A, Schweizer HP. 2006. A 10-min method for preparation of highly electrocompetent *Pseudomonas aeruginosa* cells: Application for DNA fragment transfer between chromosomes and plasmid transformation. J Microbiol Methods 64:391–397.

13. Ye X, Dong H, Huang Y-P. 2014. Highly efficient transformation of *Stenotrophomonas maltophilia* S21, an environmental isolate from soil, by electroporation. J Microbiol Methods 107:92–97.

14. Choi K-H, Mima T, Casart Y, Rholl D, Kumar A, Beacham IR, Schweizer HP. 2008. Genetic tools for select-agent-compliant manipulation of *Burkholderia pseudomallei*. Appl Environ Microbiol 74:1064–1075.

15. Choi K-H, Gaynor JB, White KG, Lopez C, Bosio CM, Karkhoff-Schweizer RR, Schweizer HP. 2005. A Tn7-based broad-range bacterial cloning and expression system. Nat Methods 2:443–448.

16. Zhao K, Tseng BS, Beckerman B, Jin F, Gibiansky ML, Harrison JJ, Luijten E, Parsek MR, Wong GCL. 2013. Psl trails guide exploration and microcolony formation in early *P. aeruginosa* biofilms. Nature 497:388–391.

17. Wick RR, Judd LM, Holt KE. 2023. Assembling the perfect bacterial genome using Oxford Nanopore and Illumina sequencing. PLOS Comput Biol 19:e1010905.

18. Wick R. 2023. Filtlong. https://github.com/rrwick/Filtlong. Retrieved 27 August 2023.

19. Wick RR, Judd LM, Cerdeira LT, Hawkey J, Méric G, Vezina B, Wyres KL, Holt KE. 2021. Trycycler: consensus long-read assemblies for bacterial genomes. Genome Biol 22:266.

20. Kolmogorov M, Yuan J, Lin Y, Pevzner PA. 2019. Assembly of long, error-prone reads using repeat graphs. 5. Nat Biotechnol 37:540–546.

21. Wick RR, Holt KE. 2021. Benchmarking of long-read assemblers for prokaryote whole genome sequencing. 8:2138. F1000Research https://doi.org/10.12688/f1000research.21782.4.

22. Vaser R, Šikić M. 2021. Time- and memory-efficient genome assembly with Raven. 5. Nat Comput Sci 1:332–336.

23. Wright C, Wykes M. 2023. Medaka. https://github.com/nanoporetech/medaka. Retrieved 28 August 2023.

24. Chen S, Zhou Y, Chen Y, Gu J. 2018. fastp: an ultra-fast all-in-one FASTQ preprocessor. Bioinformatics 34:i884–i890.

25. Wick RR, Holt KE. 2022. Polypolish: Short-read polishing of long-read bacterial genome assemblies. PLOS Comput Biol 18:e1009802.

26. Zimin AV, Salzberg SL. 2020. The genome polishing tool POLCA makes fast and accurate corrections in genome assemblies. PLOS Comput Biol 16:e1007981.

27. Aziz RK, Bartels D, Best AA, DeJongh M, Disz T, Edwards RA, Formsma K, Gerdes S, Glass EM, Kubal M, Meyer F, Olsen GJ, Olson R, Osterman AL, Overbeek RA, McNeil LK, Paarmann D, Paczian T, Parrello B, Pusch GD, Reich C, Stevens R, Vassieva O, Vonstein V, Wilke A, Zagnitko O. 2008. The RAST Server: rapid annotations using subsystems technology. BMC Genomics 9:75.

28. Overbeek R, Olson R, Pusch GD, Olsen GJ, Davis JJ, Disz T, Edwards RA, Gerdes S, Parrello B, Shukla M, Vonstein V, Wattam AR, Xia F, Stevens R. 2014. The SEED and the Rapid Annotation of microbial genomes using Subsystems Technology (RAST). Nucleic Acids Res 42:D206-214.

29. Carini P, Marsden PJ, Leff JW, Morgan EE, Strickland MS, Fierer N. 2016. Relic DNA is abundant in soil and obscures estimates of soil microbial diversity. 3. Nat Microbiol 2:1–6.

30. Lennon JT, Muscarella ME, Placella SA, Lehmkuhl BK. 2018. How, when, and where relic DNA affects microbial diversity. mBio 9:10.1128/mbio.00637-18.

31. Winder EM, Bonheyo GT. 2015. DNA Persistence in a Sink Drain Environment. PLOS ONE 10:e0134798.
